# Evaluation of the Prevalence and Risk Factors of Drug-Related Problems in Hypertension and Type 2 Diabetes Mellitus Patients at a Tertiary Care Hospital: A Cross-Sectional Study

**DOI:** 10.7759/cureus.42775

**Published:** 2023-07-31

**Authors:** Divya Reddy Peddi, Hephzibha Pallekonda, Vikas Reddy

**Affiliations:** 1 Department of Pharmacy Practice, Arya College of Pharmacy, Osmania University, Sangareddy, IND; 2 Department of General Medicine, Government District Hospital, Sangareddy, IND

**Keywords:** drug-related problems (drps), outpatients, chronic diseases, diabetes mellitus, hypertension

## Abstract

Background

Drug-related problems (DRPs) potentially interfere with the desired treatment goals which may lead to increased healthcare costs, morbidity, and mortality. Despite the negative consequences of DRPs, there is a lack of comprehensive research on their prevalence and risk factors, particularly in chronic diseases such as hypertension and type 2 diabetes mellitus (DM). This study aims to evaluate the prevalence and contributing factors of DRPs among hypertension, type 2 DM, and hypertension with type 2 DM in the outpatient general medicine department.

Methodology

A hospital-based, prospective, observational study was conducted over three months. DRPs were classified using the Helper-Strand classification. The potential risk factors contributing to DRPs were assessed using binary and multinomial logistic regression methods. A p-value <0.05 was considered statistically significant.

Results

Among the 236 study participants, DRPs were more prevalent in males, and the mean age of the participants was 51.73 ± 9.47 years. DRPs were found in 76% of the study participants, and the mean number of DRPs per patient was 1.16 ± 0.45. Among the identified DRPs, suboptimal therapeutic goals (33%) were the most frequently observed, followed by ineffective drugs (32%), medication non-adherence (23%), and drug-drug interaction (5%). Therapeutic duplication and overdose were less commonly encountered as DRPs. The presence of comorbidity (adjusted odds ratio (AOR) = 5.77), and smoking (AOR = 21.07) were found to be significant risk factors (p < 0.05) contributing to DRPs.

Conclusions

DRPs are more prevalent in hypertension, type 2 DM, and hypertension with type 2 DM. Age range (40-60 years), comorbidity, and smoking were found to be associated with a higher incidence of DRPs. The implementation of a multidisciplinary team approach involving clinical pharmacists and physicians can effectively identify the prevalence and determine the associated risk factors of DRPs and subsequently may help employ targeted interventions to mitigate the development of DRPs.

## Introduction

Uncontrolled hypertension is a major preventable risk factor for developing cardiovascular diseases, particularly ischemic heart disease, the leading cause of death worldwide [1,2]. Despite the availability of several classes of antihypertensives and lifestyle modifications, more than 80% of hypertensive patients do not have optimal control of blood pressure [3,4]. Treatment within optimal target goals, <140/80 mmHg and for patients with coexisting diabetes <130/80 mmHg, is necessary to prevent/reduce cardiovascular morbidity and mortality [5].

Type 2 diabetes mellitus (DM) is also a major leading preventable risk factor for cardiovascular diseases and mortality owing to complications such as cerebrovascular, cardiovascular, and renal diseases [6]. These complications are largely prevented/reduced when hemoglobin A1c (<7), fasting blood sugar, and postprandial blood sugar are maintained within the optimal target goals [7]. Despite the availability of several classes of oral hypoglycemics with or without insulin and lifestyle modifications, 76.8% of type 2 DM patients in India do not have optimal control of blood glucose levels [8].

The most common comorbidity in type 2 DM is hypertension, and their coexistence is associated with an increase in mortality [9,10]. Treatment of both diseases within optimal target goals is necessary to prevent cardiovascular complications. However, recent studies have shown that more than 50% of the patients with hypertension in type 2 DM do not have optimal therapeutic goals [11,12].

Antihypertensives and oral hypoglycemics with or without insulin drug therapy play a crucial role in treating and preventing complications in hypertension and type 2 DM [5,13]. However, the presence of drug-related problems (DRPs) may impact the benefits of drug therapy, increasing healthcare costs, significant morbidity, and mortality [14]. DRPs are vastly increasing in chronic diseases [15].

A DRP is defined as an event or circumstance that involves drug therapy that potentially interferes with a desired patient outcome or goal [16]. Mostly, these are preventable with the right intervention of the healthcare system [17].

To our knowledge, more extensive studies have been conducted on the prevalence of uncontrolled hypertension and uncontrolled DM [1,8]. However, there are scarce studies conducted on the prevalence and contributing factors of DRPs, particularly in chronic diseases such as hypertension and DM. This study aims to assess the prevalence and contributing factors of DRPs in hypertension, type 2 DM, and hypertension with type 2 DM.

## Materials and methods

Study site and study design

A hospital-based, prospective, observational study was conducted at the ambulatory general medicine department of Sangareddy District Government Hospital (SDGH), Telangana. SDGH is a teaching and tertiary care hospital. It is one of Telangana’s largest public hospitals, estimated to provide healthcare to more than 4.4 million people. This study was approved by the institutional ethics committee of Arya College of Pharmacy, Osmania University (approval number: 1920212201).

Study participants and sample size

Patients visiting the hospital for refilling their medications during the study period of January to March 2022 were screened for eligibility and included in this study. Patients aged above 18 years with hypertension, type 2 DM, and hypertension with type 2 DM were included in the study. Patients with any other comorbidities (e.g., hypo/hyperthyroidism, heart failure), gestational DM, gestational hypertension, and mental instability were excluded from the study.

A sample size of 384 was calculated using the sample proportion formula with the assumption of a 5% margin of error, 95% confidence interval (CI), and 50% prevalence of DRPs. We approached a total of 384 patients who fulfilled the inclusion criteria but only 236 agreed to participate in this study. Written informed consent was obtained from all patients.

Data collection

A data abstraction form was used to collect demographic details and medication history. Demographic details included age, gender, smoking history, and family history of the study participants. Medication history including dose, dosage form, drug name, and medication adherence was collected in the above-mentioned form. Blood pressure was measured manually twice on different days, and fasting blood sugar, postprandial blood sugar, hemoglobin A1c, fasting lipid panel, and other laboratory measurements were documented.

We used the Helper and Strand classification of DRPs to identify DRPs in hypertension, type 2 DM, and hypertension with type 2 DM [18]. Adverse drug reactions were assessed using the Naranjo algorithm scale [19]. Medication adherence was measured using the proportion of days covered [20]. Apart from Helper and Strand classification, we used a few other drug-related problems, i.e., therapeutic duplication, medication non-adherence, and the need for additional drug therapy. A total of three physicians, four clinical pharmacists, two nurses, and two laboratory technicians were involved in this study.

Helper and Strand classified DRPs based on their potential to interfere with the therapeutic outcomes. These DRPs were classified into the following eight categories:

1. Ineffective drug: the least effective drug is chosen despite the availability of a more effective alternative.

2. Untreated indication: the patient has a medical condition that necessitates drug therapy but is not receiving the drug for it.

3. Subtherapeutic optimal goal: the DRP includes a low dose (a drug given in a low dose) and needs additional new drug therapy (combination therapy is required to maintain the therapeutic goal).

4. Medication non-adherence: patient not taking drugs as prescribed.

5. Drug-drug interaction: a patient’s medical condition requires a combination of two drugs that have the potential to interact with each other.

6. Adverse drug reaction: an adverse effect refers to a negative outcome that arises from the use of a drug.

7. Therapeutic duplication: therapeutic duplication involves the prescription and dispensing of the same drug or two or more drugs from the same therapeutic class.

8. Overdose: patient taking drugs in a high dose.

Statistical analysis

We entered the categorical and continuous data into the SPSS software (IBM Corp., Armonk, NY, USA). A careful review and analysis were done by clinical pharmacists. Descriptive statistics were used to assess the mean, standard deviation, and frequencies of the categorical and continuous variables. The binary and multinomial logistic regression method was used to assess the relationship between the dependent variable (DRPs) and independent variables (age, gender, smoking history, family history, and comorbidities).

## Results

Patient demographics

A total of 384 patients were reviewed, of whom 236 were included in the study. Of the total, the majority of the study participants were males (54%), and the mean age of the study participants was 51.73 ± 9.47 years (Table 1). The majority of the male study participants were smokers. All female participants were non-smokers. Overall, 126 (53%) participants had hypertension with type 2 DM.

**Table 1:** Demographic characteristics of the study participants (n = 236).

Patient characteristics	Category	n (%)
Age (year)	30–39	16 (7%)
	40–49	80 (34%)
	50–59	73 (31%)
	60–69	57 (24%)
	70–79	10 (4%)
Gender	Male	127 (54%)
	Female	109 (46%)
Family history	Presence	65 (28%)
	Absence	171 (72%)
Smoking history	Smokers (males)	77 (33%)
	Non-smokers (males and females)	159 (67%)
Disease	Hypertension	61 (26%)
	Type 2 diabetes mellitus	49 (21%)
	Hypertension with type 2 diabetes mellitus	126 (53%)

Number of drug-related problems

**Figure 1:**
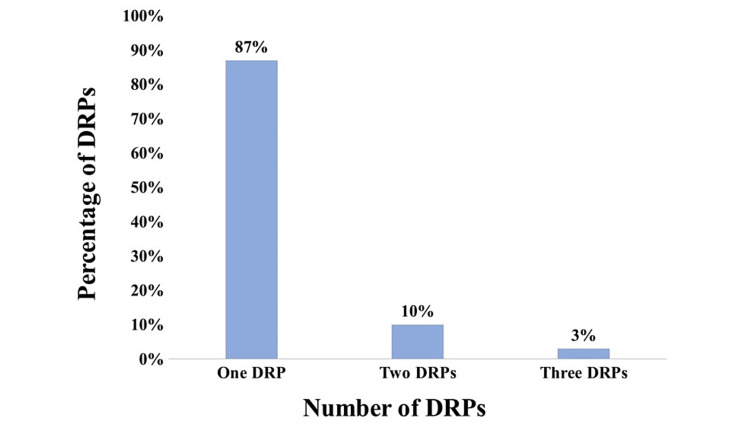
Number of drug-related problems (DRPs).

A total of 181 patients were found to have at least one/more DRPs. Out of the total, 211 DRPs were identified, with a mean DRP per patient of 1.16 ± 0.45. One DRP was identified in 157 (87%) patients, and two and three DRPs were found in 18 (10%) and 6 (3%) of the patients, respectively, as shown in Figure 1.

Types of drug-related problems

Of the 181 patients, 211 DRPs were identified. The frequently identified DRPs were suboptimal therapeutic goals (33.6%), ineffective drug (32%), and medication non-adherence (23%), followed by drug-drug interactions (5%). The less commonly identified DRPs were untreated indication (3%), followed by adverse drug reaction (2%), and therapeutic duplication (1%) (Table 2).

**Table 2:** Category of drug-related problems (n = 211).

Category	n (%)
Subtherapeutic goal/Clinical inertia	71 (33.6%)
Ineffective drug	67 (32%)
Medication non-adherence	48 (23%)
Drug-drug interaction	12 (5%)
Untreated indication	6 (3%)
Adverse drug reaction	4 (2%)
Therapeutic duplication	2 (1%)
Overdose	1 (0.4%)

Factors to determine predictors of drug-related problems

Binary and multinomial logistic regression methods were used to determine the predictors of DRPs. Gender, age, smoking history, and comorbidities were the independent variables for DRPs. Comorbidities and smoking history (p < 0.001) were contributing factors in the occurrence of DRPs (Table 3). The likelihood of having DPRs was about five times higher in patients who had comorbidity (adjusted odds ratio (AOR) = 5.77, 95% CI = 2.76-12.04). Patients who were smokers were 20 times more likely to have DRPs compared to non-smokers (AOR = 21.07, 95% CI = 5.45-81.47).

**Table 3:** Determination of factors contributing to the occurrence of drug-related problems (DRPs).

Independent variables	Category	DRPs		Crude odds ratio	Significance	Adjusted odds ratio	Significance
		Yes	No				
Age (year)	≤45	57	20				
	46–60	94	24	1.37 (0.70-2.70)	0.36	0.93 (0.43-2.03)	0.85
	>60	30	11	0.96 (0.41-2.26)	0.92	0.88 (0.32-2.41)	0.81
Smoking	No	107	52	11.99 (3.61-39.84)	<0.001	21.07 (5.45-81.47)	<0.001
	Yes	74	3				
Comorbidity	No	69	41	4.75 (2.42-9.35)	<0.001	5.77 (2.76-12.04)	<0.001
	Yes	112	14				
Gender, reference: female	109	77	32	1.88 (1.02-3.46)	0.043	0.53 (0.24-1.17)	0.12

## Discussion

Chronic diseases have a higher prevalence of DRPs due to comorbidities, polypharmacy, and medication non-adherence [15]. DRPs potentially interfere with the desired patient goals in hypertension and type 2 DM and may lead to macrovascular (cardiovascular) and microvascular complications that significantly increase morbidity and mortality [16,21,22]. Analyzing the occurrence of DRPs and their prevention reduces morbidity and healthcare-associated costs [23].

DRPs were identified in 76% of the study participants, and the mean number of DRPs per patient was 1.16 ± 0.45. To date, this is the first study conducted on DRPs among all three diseases, i.e., hypertension, type 2 DM, and hypertension with type 2 DM. In contrast to studies conducted in Indonesia and Ethiopia, DRPs in hypertension were found in 57% of the patients, which is lower than our study, and DRPs in type 2 DM and hypertension with type 2 DM were found in 83.1% (1.8 ± 0.751) and 86% (1.65 ± 1.05 per patient), respectively, which is higher than our study [24-26]. This variation might be due to population differences, as our study included different chronic diseases, clinical characteristics, population demographics, and methods used to classify DRPs.

In accordance with the studies conducted in Ethiopia and Spain, the frequently identified DRP was found to be a suboptimal therapeutic goal (need for additional drug therapy/dose too low), followed by ineffective drug and medication non-adherence (25,26). In contrast to the study conducted in Indonesia on hypertension, the most common DRPs were adverse drug events and untreated indications [24]. This variation might be due to population demographics, medical comorbidities, and polypharmacy.

The increased predominance of suboptimal therapeutic goals might be due to the presence of comorbidities, hypertension with type 2 DM, and a lack of regular screening/monitoring for hypertension and type 2 DM patients. The ineffective drug may be due to a lack of knowledge of patient comorbidities. For instance, patients who were diagnosed with both hypertension and type 2 DM were treated with calcium channel blockers, whereas guidelines suggest that they should be treated with angiotensin-converting enzyme inhibitors or angiotensin receptor blockers because of nephroprotective action. Medication non-adherence was also commonly identified as a DRP which may be due to a lack of importance of medication, poor memory, and false beliefs, among others.

The majority of DRPs were found in the age group of 40-60 years, and the mean age of the study participants was 51.73 ± 9.47 years, which is approximately consistent with the study conducted by Yimama et al. where the mean age was 54.44 ± 11.68 years [26]. The highest DRPs among these age groups may be suggestive of the increased prevalence of hypertension with type 2 DM comorbidity among these patients [27]. Consistent with the study conducted by Yimama et al., the prevalence of DRPs was higher in males than in females, indicating a gender-based difference [26]. It is possible that smoking, higher incidence of hypertension, and type 2 DM among males contribute to such differences. We observed an increased occurrence of DRPs among patients who were smokers compared to non-smokers, and the difference was statistically significant (p < 0.001). In our study, males had higher DRPs because smoking is a confounder that affects the outcome associated with blood pressure and blood glucose level fluctuations, necessitating the need for additional drug therapy to attain target therapeutic goals [28,29].

In accordance with the study from Ethiopia, our data demonstrated that the presence of comorbidity, i.e., hypertension with type 2 DM, significantly(p < 0.001) increases the occurrence of DRPs [30]. Many studies have shown that achieving the desired therapeutic outcome for individuals with type 2 DM and comorbid hypertension through monotherapy is challenging, resulting in suboptimal results. Consequently, they often necessitate multiple drugs, which could be a contributing factor to the rise in DRPs [12,27].

This study has some limitations. DRPs were not classified based on the severity into mild, moderate, and severe. The outcomes of this research may be influenced by the demographics of the population and the methods employed for identifying and categorizing DRPs.

## Conclusions

DRPs are more prevalent in hypertension, type 2 DM, and hypertension with type 2 DM. DRPs increase healthcare costs, morbidity, and mortality. Age range (40-60 years), comorbidity, and smoking are associated with a higher incidence of DRPs. The implementation of a multidisciplinary team approach involving clinical pharmacists and physicians can effectively identify the prevalence and determine the associated risk factors of DRPs and may help employ targeted interventions to mitigate the development of DRPs.
